# Acute Brucellosis and Cirrhosis: The Triggering Event of Fatal Liver Decompensation

**DOI:** 10.1155/2020/8868001

**Published:** 2020-12-22

**Authors:** Maria Kosmidou, Eleftherios Klouras, Iro Rapti, Sebastien Filippas-Ntekouan, Haralampos Milionis

**Affiliations:** First Division of Internal Medicine, Faculty of Medicine, University of Ioannina, Ioannina, Greece

## Abstract

Cirrhotic patients are known to be particularly susceptible to infectious complications that may vary according to regional endemic patterns. Brucellosis, a common zoonosis with worldwide distribution, exhibits a predilection for the reticuloendothelial system and thus resulting in hepatic involvement. We describe three cirrhotic patients in whom acute brucellosis and/or its treatment served as the triggering factor of hepatic decompensation, with deleterious effects. The patients suffered from alcoholic cirrhosis and culture-proven brucellosis. All patients came from an area endemic to brucellosis. The first patient exhibited a relapsing brucellosis course with progressive deterioration of his fragile liver function. The second patient progressed rapidly to jaundice, possibly partly attributed to antibiotic pharmacotoxicity, and died during liver transplantation. The third patient eventually succumbed to diffuse intravascular coagulation. Brucellosis can be a triggering event of fatal liver decompensation in cirrhotic patients. Enhancing health literacy of the patients, particularly in endemic areas, is of paramount importance for prevention of exposure to similar pathogens.

## 1. Introduction

Brucellosis, one of the most ubiquitous zoonotic infections worldwide [1], can induce a wide spectrum of clinical complications that range from mild, not necessitating alteration of antibiotic regimen synthesis or duration, to fortunately rare, fatal cases typically attributed to cardiac or central nervous system involvement [1]. Increasing reports incriminate brucellosis as a triggering factor on overall health derangement in patients with underlying diseases, typically as an opportunistic infection in patients with hematologic [2] or rheumatologic disorders [3]. The liver, serving as the largest organ of the reticuloendothelial system (RES) for which *Brucella* exhibits a typical predisposition, is usually affected in acute brucellosis in the form of mild biochemical alterations and/or hepatomegaly in a percentage of patients [4]. The presence of hepatic granulomas, disputed in the past in the case of the commonest species, *B. melitensis*, has been ascertained in recent years [5], although not necessarily indicating an adverse outcome. On the contrary, chronic hepatic suppurative disease remains one of the rare but therapeutically challenging forms of chronic brucellosis [6]. An etiological role of brucellosis in cirrhosis development had been supported in the early 1950s by pioneers of *Brucella* research such as Wesley Spink [7]. But, this correlation gradually regressed to isolated case reports of doubtful significance, in subsequent years. In cases of preexisting liver disease, numerous case reports have demonstrated brucellosis as a causative factor of disequilibrium development, characteristically in the form of ascites/spontaneous bacterial peritonitis (SBP) development [8–16].

Given that cirrhotic patients, in general, are predisposed through a variety of pathogenic immune disorder mechanisms to infection development, not only restricted to SBP [17], it is of paramount importance to increase these patients' health literacy in order to prevent opportunistic infections from uncommon, endemic in specific areas, pathogens such as *Vibrio vulnificus* in Southest Asia [18] or brucellosis in numerous parts of the world. The last is of further significance due to its need of protracted combined antibiotic therapy with regimens exhibiting acknowledged potential of hepatotoxicity.

Herein, we describe three cases of cirrhotic patients where acute brucellosis served as a trigger of fatal hepatic decompensation, either due to direct effect of the pathogen alone, or in combination with hepatotoxicity of the necessary antibiotic regiments used. We further underline the necessity, in face of these specific therapeutic difficulties, of raising awareness of cirrhotic patients in terms of avoidance of exposure to such pathogens.

## 2. Case Presentations

### 2.1. Patient 1

A 47-year-old male, with known alcoholic cirrhosis for the past 6 years, was admitted for persisting evening fever and abdominal dilatation. The patient abstained from alcohol since diagnosis and had a history of esophageal variceal bleeding, hepatic encephalopathy episodes, and ascites development in the past but had been ascites-free for at least 6 months prior to admission. His medical history also included insulin-dependent diabetes mellitus. He continued to work as a confectioner. On admission, clinical evaluation and subsequent imaging demonstrated massive ascites and splenomegaly, with no signs of portal thrombosis. Laboratory evaluation showed mild pancytopenia and increase C-reactive protein (CRP), while other biochemistry markers were, in general, normal. Numerous cultures were drawn from multiple sites, including bone marrow, and serology tests for various pathogens were performed. Cultures were positive for *B. melitensis*, a diagnosis further confirmed by real time-polymerase chain reaction (PCR based on direct amplification of a 207-base pair DNA sequence of a gene specific for the *Brucella* genus, BCSP31) and agglutination (Rose Bengal plate agglutination test), Wright seroagglutination test, and ELISA serology (Serion ELISA IgG/IgM/IgA antibodies to *Brucella melitensis)*. The patient retrospectively admitted to consuming unpasteurized dairy products (sheep and goats) 2 months prior to admission.

Initiation of antibiotic therapy was problematic since there was a vague history of tetracycline intolerance in the past, and the underlying pathology advocated against the use of the hepatotoxic doxycycline and rifampicin and the potentially hepatorenal syndrome inducers, aminoglycosides. The remaining options were trimethoprim/sulfamethoxazole 160/800 mg twice daily and ciprofloxacin 500 mg twice daily, which were initiated as soon as diagnosis was confirmed. Defervescence was indeed achieved on day 5, albeit accompanied by renal function disorders, with a gradual increase in serum creatinine up to 2 mg/dl, with normal values (nv) of 0.6–1.2 mg/dl. On day 12, fever relapsed, accompanied by worsening pancytopenia and renal function values. Extensive diagnostic workup was performed, including further cultures and imaging and excluding any other underlying infection, and it was decided to substitute cotrimoxazole with doxycycline 100 mg twice daily, since the benefit of brucellosis therapy overruled the risk of doxycycline-hepatotoxicity at that point. Defervescence was achieved 8 days later, accompanied by partial reversal of renal function and pancytopenia. The patient was successfully discharged and referred to a transplantation center for further evaluation. Forty days after doxycycline-ciprofloxacin initiation, the patient was readmitted due to hepatic encephalopathy, worsening of ascites and pancytopenia, and low-grade fever: any underlying cause was excluded through a scrutinized workup and supportive measures initiation. Doxycycline was discontinued as a potential inducer of decompensation, and the patient was transferred to a transplantation center.

Since pulmonary hypertension had been detected, the patient was not considered a candidate for liver transplantation and he continued to rapidly deteriorate until the adverse outcome.

### 2.2. Patient 2

A 50-year-old man, with diagnosed alcoholic cirrhosis for the past decade, abstaining from alcohol since then, with no history of any cirrhotic complications, under no supportive treatment apart for regular follow-up, was admitted for protracted fever of two months' duration, accompanied by low backache, mild diarrhea, and abdominal distention. He had a history of splenectomy and was a shepherd. Significant ascites, accompanied by moderate pleural fluid effusion, was noted on clinical examination and subsequent imaging, the latter also demonstrating abdominal lymphadenopathy with partial central necrosis. Laboratory tests, compared to his latest follow-up, demonstrated only an increase in CRP and total and direct bilirubin. Blood cultures were positive for *B. melitensis*, a finding further confirmed by Rose Bengal plate agglutination, Wright seroagglutination test, and ELISA serology (Serion ELISA IgG/IgM/IgA antibodies to *Brucella melitensis*). The patient was treated with doxycycline 100 mg twice daily, rifampicin 600 mg daily, and ciprofloxacin 500 mg twice daily. Defervescence was achieved on day 5, accompanied though by progressive worsening of liver biochemistry indices and mild prolongation of international normalized ratio (INR) to levels of 1.34 (nv 0.8–1.2). Rifampicin dose was halved, and diuretics were added to treat ascites. Despite improvement/stabilization of other biochemical indices in later days, serum bilirubin continued to increase, reaching a plateau of total 7.2 mg/dl/direct 4.2 mg/dl and the patient was discharged, continuing treatment and follow-up as an outpatient on day 16. On day 41, the patient was readmitted with hepatic encephalopathy and jaundice. Hepatic biochemistry was steady, but total bilirubin was 16.7 mg/dl (direct bilirubin 13.6 mg/dl) and INR was 1.44. Ascites was minimal and subsequent workup excluded any underlying causes of encephalopathy. Antibiotics were discontinued as potential inducers of severe hepatotoxicity, and the patient was referred to a transplantation center due to the gradual worsening of liver function indices. He underwent liver transplantation which unfortunately had an adverse postoperative outcome.

### 2.3. Patient 3

A 46-year-old male with a long history of alcohol abuse was transferred to our department with a novel diagnosis of alcoholic cirrhosis. He had been admitted to another hospital with delirium tremens and rhabdomyolysis. A diagnosis of alcoholic cirrhosis was made following evaluation. During that hospitalization, mild fever was observed, which was considered as a consequence of aspiration and treated with amoxicillin-clavulanate. During hospitalization to our department for further evaluation of cirrhosis (which demonstrated extensive ascites, splenomegaly, prolongation of international normalized ratio (INR), hypoalbuminemia, and mild lower esophageal varices), low-grade fever persisted. Medical history revealed a recent, 50 days ago, trip to Spain and consumption of nonpasteurized dairy products (sheep's products).

Blood cultures were positive for *B. melitensis*, confirmed by Rose Bengal plate agglutination, Wright seroagglutination test, and ELISA serology (Serion ELISA IgG/IgM/IgA antibodies to *Brucella melitensis*). The patient was treated with doxycycline 100 mg twice daily and ciprofloxacin 500 mg twice daily, but on day 4, he exhibited rapid worsening of liver function and laboratory indices of diffuse intravascular coagulation (DIC). Discontinuation of doxycycline and subsequently ciprofloxacin, along with supportive treatment, did not reverse the rapid evolution of the syndrome towards hepatic encephalopathy, spontaneous intracranial hemorrhage, and death on day 33.

## 3. Discussion

In recent years, brucellosis has been recognized as a significant cause of morbidity in the developing world and also as an important parameter of travel-related medicine [1]. Numerous reports have demonstrated the emergence of disease in the developed world not only through international travel but also through dairy products' trade and immigration [1, 19]. Awareness of the disease remains limited even in regions of hyperendemicity [20]. All patients in this series were located in an endemic area from an endemic country; yet, even though employed in theoretically high-risk occupation, the first two patients did not avoid exposure through consumption of unpasteurized dairy products or direct contact, respectively. The third patient, on the other hand, is a typical example of travel-related brucellosis.

Hepatic complications are expected in brucellosis, since the liver is the largest RES organ in the human body. One should not forget that the peritoneal mononuclear phagocytic system is an integral part of RES and so it is thus not surprising that it can react by ascites formation even in previously healthy patients [21, 22], or in the context of generalized fulminant disease course [23], let alone in cirrhosis.

Treating brucellosis has always been problematic due to the pathogen's ability to survive inside human phagocytic cells and trigger relapses that can be observed even when adequate therapy is administered. Both the World Health Organization and expert therapeutic recommendations [24] stress the need for a combined protracted therapeutic regimen. In cases of preexisting liver disease though, concerns about the toxicity of the antibacterial agents active against brucellosis are further enhanced due to the need of protracted administration. Using doxycycline 100 mg twice daily and rifampicin 600 mg or 900 mg once daily for six weeks at least, and even more in cases of relapse or serious complications as spondylitis, carries an expectedly increased potential for pharmaceutical toxicity. On the other hand, using amynoglycosides in patients who have already developed cirrhotic ascites may predispose to hepatorenal syndrome and generally endanger an intravascular/extravascular volume equilibrium that is often extremely difficult to achieve in cirrhotic patients. Thus, it is difficult to administer any of the three accepted prime combinations in patients with cirrhosis and portal hypertension complications, such as doxycycline with either streptomycin or rifampicin or gentamycin. This is characteristically observed in the second patient, where the administration of both doxycycline and rifampicin may have played an important role in liver decompensation. Relevant literature on *Brucella* and SBP includes reports of successful therapy with minimal doses of rifampicin, but conclusions cannot be extrapolated from isolated cases, particularly since susceptibility of each cirrhotic patient to pharmaceutical hepatic damage cannot be prequantified. The present study focuses only on patients with an adverse outcome and does not include patients with cirrhosis, who were successfully treated with typical brucellosis regimens in our department. Furthermore, the severity of each brucellosis case is not similar, and the above-reported cases were acute, culture-positive brucellosis cases that satisfied all accepted predisposing factors to treatment relapse and failure [25]. Indeed, similar published experience varies largely regarding the outcome, since there have been reports of successful monotherapy with ofloxacin [8], of successful use of minimal dose of rifampicin with either doxycycline or ciprofloxacin [9, 10], and of prolonged courses of doxycycline for concurrent spondylitis or chronic hepatosuppurative disease [11, 12]. On the other hand, there is also published experience of adverse outcomes, similar to this series [13], with a report of interest being strikingly similar to the third patient described in the present study, with rapid deterioration and DIC development [14].

This brings us back to prevention, particularly through increasing health literacy of the patients and more specifically by acknowledging individual risk factors related to region of residence, occupation, other underlying diseases, or cause of cirrhosis [17]. Zoonoses should not be forgotten, given their global distribution and therapeutic peculiarities.

## Data Availability

No data were used to support this study.
